# Corino de Andrade disease: mechanisms and impact on reproduction

**DOI:** 10.5935/1518-0557.20170025

**Published:** 2017

**Authors:** Rita A Lopes, Teresa Coelho, Alberto Barros, Mário Sousa

**Affiliations:** 1 Laboratory of Cell Biology, Department of Microscopy, Institute of Biomedical Sciences Abel Salazar (ICBAS), University of Porto, Portugal; 2 Department of Neurophysiology, Research Center of Corino de Andrade (Paramyloidosis), Hospital Centre of Porto, Portugal; 3 Centre for Reproductive Genetics Prof. Alberto Barros (CGR), Porto, Portugal; 4 Department of Genetics - School of Medicine, Institute of Health Research and Innovation, University of Porto

**Keywords:** Transthyretin-related hereditary amyloidosis, physiopathology, genetics, sexual dysfunction, therapy, in vitro fertilization

## Abstract

Familial amyloid polyneuropathy was first described by Corino de Andrade in 1952 in Northern Portugal. It is a fatal autosomal dominant neurodegenerative disorder characterized by a progression of neurologic symptoms, beginning early in the reproductive life. The Transthyretin gene mutation originates a mutated protein that precipitates in the connective tissue as amyloid deposits. This disease is presently named Transthyretin-related hereditary amyloidosis. We performed an extensive review on this disease based on searches in Medical databases and in paper references. In this review, we briefly summarize the epidemiology and the mechanisms involved on amyloid deposition; we detailed how to evaluate the mechanisms implicated on the development of the major signs and symptoms associated with reproductive dysfunction; and we discuss the mechanisms involved in secondary sexual dysfunction after psychological treatments. Treatment of the disease is directed towards relieving specific symptoms in association with liver transplant, and molecular and genetic therapeutics. Although the current clinical trials indicate symptoms relief, no data on the reproductive function was reported. Thus, preimplantation genetic diagnosis is presently the only available technique that eradicates the disease as it avoids the birth of new patients.

## INTRODUCTION

Familial amyloid polyneuropathy was first described in 1952 by Corino de Andrade in Northern Portugal (Andrade, 1952); being presently named Transthyretin-related hereditary amyloidosis (V30M). It is a chronic fatal hereditary autosomal dominant neurodegenerative disorder, with a high prevalence in that endemic region (1:1000). The progressive sensory, autonomic and motor neuropathies eventually leads to cachexia and/or cardiovascular collapse, and results in death 10-20 years after the onset of symptoms (Sousa *et al.*, 1995; Ando *et al.*, 2013). It is caused by a single nucleotide mutation in the Transthyretin (TTR) gene (Saraiva *et al.*, 1984; Benson & Kincaid, 2007).

Most of the affected individuals are heterozygous and thus express both normal and variant TTR. Although carriers of the mutation have a circulating mutant protein since fetal life, no toxic amyloid deposition occurs until adulthood. This disease is thus mainly diagnosed in young adults (high penetrance) with less than 40 years of age (early-onset), at a similar gender-wise ratio. In these patients, 60% develop symptoms between 25-35 years and 87% before 40 years of age (Saraiva *et al.*, 1984; Benson & Kincaid, 2007; Ando *et al.*, 2013). In regions outside the endemic area, the V30M mutation has a late-onset (after 50 years of age) of symptoms (reduced penetrance) and some individuals may remain asymptomatic for life. Patients are thus frequently diagnosed after having achieved a natural conception (Sousa *et al.*, 1995; Coelho *et al.*, 1994; Soares *et al.*, 1999, 2004, 2005).

### 1. Molecular mechanisms involved in amyloid deposition, toxic effects and tissue specificity

The gene that codes for the TTR protein is located on chromosome 18. Transthyretin is the plasma transport protein for thyroid hormone thyroxin and retinol-binding protein/vitamin A. It is made of four monomers that associate into dimers and then into a tetramer. Although mainly produced by hepatocytes, it is also synthesized in the retinal pigment epithelium of the eye and in the ventricular choroid plexus of the brain. The TTR protein does not cross the blood-brain barrier (BBB) and the concentrations found in the cerebrospinal fluid (CSF) are much higher than those in the plasma (Saraiva *et al.*, 1984, 1985; Coelho *et al.*, 1994; Hou *et al.*, 2007; Teixeira *et al.*, 2013).

**Figure 1:**
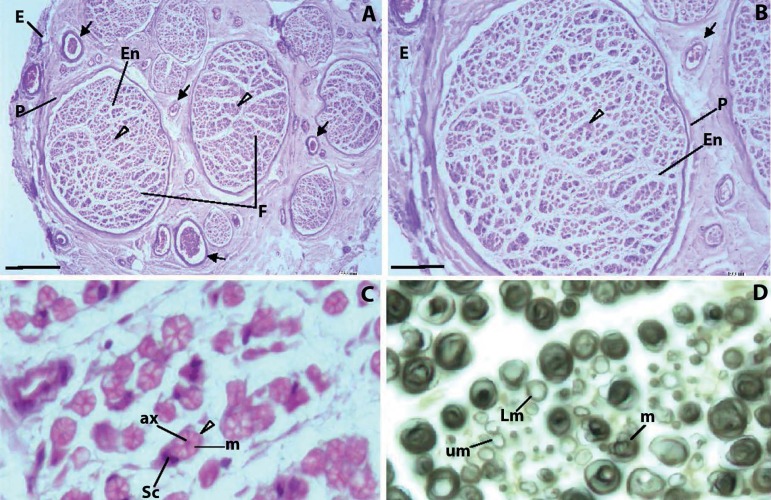
Typical appearance of peripheral nerves. A, B. Each nerve is surrounded by a dense connective tissue, the epineurium (E). Nerves are a group of nerve fibers (arrowheads) disposed into different size aggregates - the nerve fascicles (F). Nerve bundles are separated by loose connective tissue, the perineurium (P). Numerous blood vessels (arrows) can be seen in the perineurium. Each bundle presents numerous axons surrounded by loose connective tissue, the endoneurium (En). In myelinated axons, Schwann cells develop thin cytoplasmic extensions that wrap over the axon surface, creating the myelin sheath. The cytoplasmic membrane of the Schwann cell is tightly connected to the axon cytoplasmic membrane, controlling axon metabolism through transmembrane channels and pH, providing the ATP necessary for kinesin-continued transport of neurotransmitter vesicles along microtubules, and scavenging free toxic oxygen radicals produced during electric impulses. The myelin layer is a barrier to most molecules, and only very small liposoluble molecules can traverse the myelin sheath. C. In myelinated axons, each nerve fibber consists of an axon (ax) surrounded by the myelin sheath (m) of Shawn cells (Sc). Unmyelinated axons are nevertheless surrounded by Schwann cells. D. Myelinated fibers (m), light myelinated fibers (Lm) and unmyelinated fibers (um). A-C. Sural nerve, Hemalumen-eosin. D. Sciatic nerve, Osmic acid. Bars: A: 200 µm; B: 100 µm; C: 9 µm; D: 9 µm.

The mutated TTR is originated from a missense point mutation in exon 2, that causes the replacement of amino acid valine for methionine at position 30. This causes a conformational change whose instability facilitates the dissociation of the tetramer into monomers. Monomers then precipitate in the loose connective tissue and progressively self-assemble into amyloid fibrils (Costa *et al.*, 1978; Saraiva *et al.*, 1984, 1985; Benson & Kincaid, 2007; Hou *et al.*, 2007; Planté-Bordeneuve & Said, 2011; Gasperini & Small, 2012; Teixeira *et al.*, 2013). Precipitated monomers interact with the cytoplasmic membrane of Schwann cells (Figure 1), causing calcium influx, and this intracellular increase in calcium induces the release of further calcium from calcium stores. The high level of intracellular calcium is toxic and promotes an increase in free oxygen radical concentration, which then causes membrane lipid peroxidation. This is followed by the activation of apoptosis mechanisms. Additionally, abnormal TTR also precipitates in the loose connective tissue of endoneurium capillaries (Figure 1), causing similar injury cell events. Injured Schwann cells and endothelial cells then activate an inflammatory response. All the phenomena lead to progressive demyelination and neuron loss, which is aggravated by nerve ischemia due to a compression effect (Sousa *et al.*, 2001, 2004; Teixeira *et al.*, 2006; Benson & Kincaid, 2007; Hou *et al.*, 2007; Planté-Bordeneuve & Said, 2011; Gasperini & Small, 2012).

The disease initiates in the peripheral nervous system (PNS). The mutated protein in the PNS originates in the circulation and by CFS diffusion. Since the blood-nerve barrier (BNB) is much weaker than the BBB, TTR can easily traverse the BNB (Sousa *et al.*, 2004). In addition, TTR expression was also found in Schwann cells, which would enable PNS direct attainment (Murakami *et al.*, 2010). It is suspected that cells more resistant to amyloid deposition are surrounded by less connective tissue and/or have larger defense mechanisms, as in hepatocytes, the main site of TTR synthesis. On the contrary, axons are surrounded by large amounts of loose connective tissue and have poor defense mechanisms. The beginning of symptoms in the body extremities can thus be explained by the fact that these axons are very long and distant from the neural cell bodies (lack of the appropriate antioxidant systems needed to defend against peroxide membrane damage) and present more small myelinated and unmyelinated fibers (less myelin protection) (Sousa *et al.*, 2004).

### 2. Molecular mechanisms of the disease

V30M affects both branches of the PNS, somatic and autonomic, with different nerve fibers being affected at different stages of the disease. In the early stages, the smaller nerve fibers (small myelinated and unmyelinated), which mediate pain, thermal sensation and autonomic function, are affected. As the disease moves on to more advanced stages, the larger myelinated fibers (large nerve fibers are heavily myelinated and mediate motor strength, vibratory and touch sensation) start being impaired and destroyed. As the function of the limbs deteriorates, the autonomic nervous system also becomes impaired by amyloid deposition. Early symptoms correspond to neurogenic digestive disturbances. With time, patients refer that all they eat is immediately evacuated, which leads to cachexia (Andrade, 1952; Coelho *et al.*, 1994; Benson & Kincaid, 2007; Hou *et al.*, 2007; Planté-Bordeneuve & Said, 2011; Ando *et al.*, 2013).

Besides peripheral neuropathy and gastrointestinal impairment, other clinical problems originate from V30M. Orthostatic hypotension (Benson & Kincaid, 2007; Planté-Bordeneuve & Said, 2011; Ando *et al.*, 2013), Nephropathy (Lobato *et al.*, 2003; Benson & Kincaid, 2007; Lobato & Rocha, 2012; Planté-Bordeneuve & Said, 2011; Ando *et al.*, 2013), Ocular diseases (Benson & Kincaid, 2007; Cunha-Vaz, 2009; Beirão *et al.*, 2012, 2013; Planté-Bordeneuve & Said, 2011; Ando *et al.*, 2013), Heart diseases (Benson & Kincaid, 2007; Planté-Bordeneuve & Said, 2011; Ando *et al.*, 2013), and reproductive problems.

### 3. Problems related to the reproductive system

**Figure 2:**
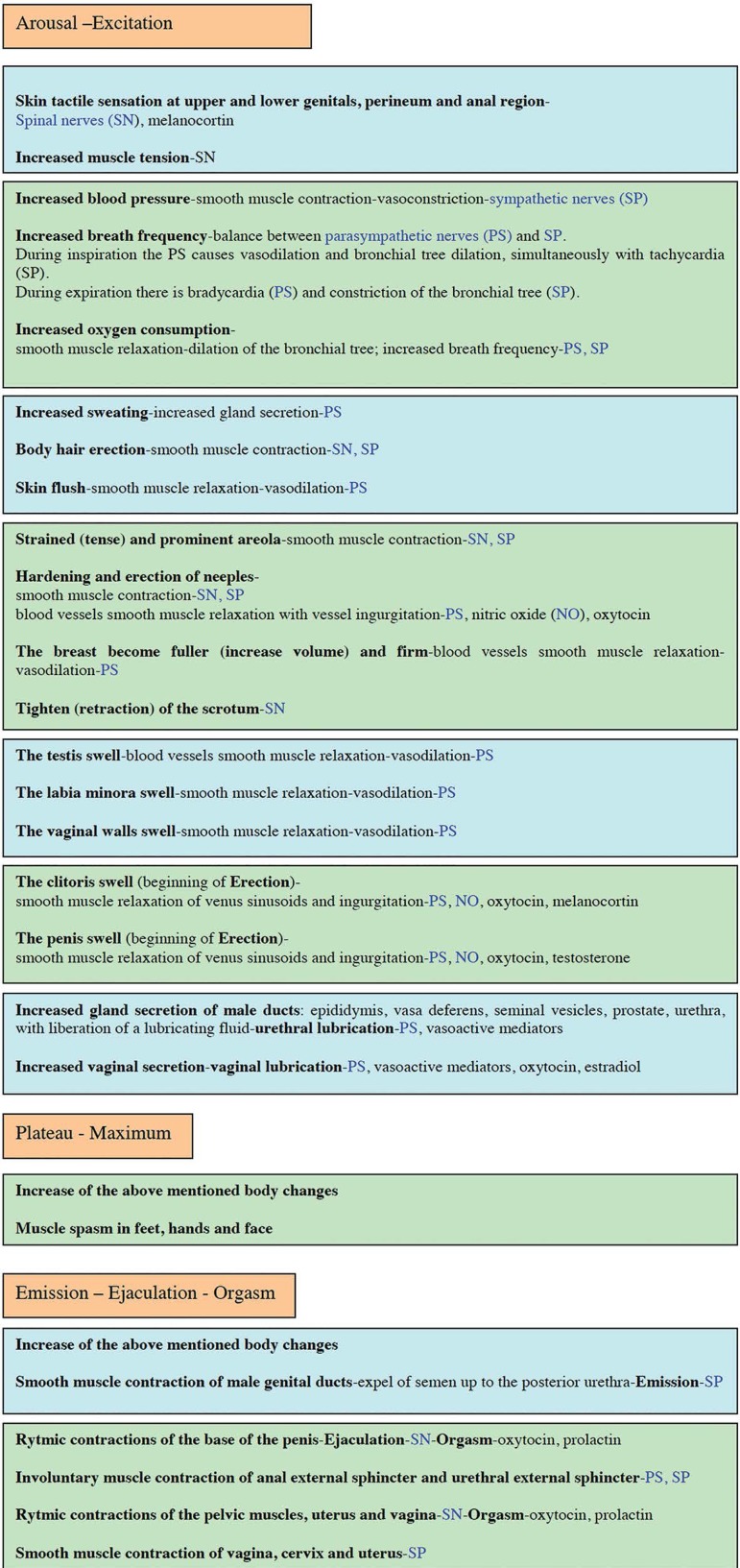
Phases of the sexual response. Control mechanisms by the peripheral nervous system, somatic and autonomic (sympathetic and parasympathetic).

Patients with V30M develop sexual impairments due to psychological and physical problems, with the latter being related to peripheral nerve loss and vascular ischemia. Sexual responses (Figure 2) are mediated by the coordinated action of the PNS: sympathetic, parasympathetic and somatic sensory nerves. They have two synapses. The first synapse links preganglionic neurons (in the spine) to postganglionic neurons (adjacent to the spine), which is mediated by the neurotransmitter acetylcholine that acts over nicotinic receptors. The second synapse links postganglionic neurons to the target tissue, being mediated by neurotransmitters norepinephrine/noradrenaline (or epinephrine/adrenaline), that act on adrenergic receptors. Parasympathetic nerves have their postganglionic ganglia in the pelvis. The neurotransmitter is acetylcholine, that acts on cholinergic receptors in target tissues. Vascular arterial dilation (penis and clitoris erection) depend of parasympathetic nerves, with a simultaneous relaxation of smooth muscles of the venous (cavernous) sinusoids by means of nitric oxide. The parasympathetic nerves also stimulate the vas deferens, seminal vesicles, prostate and vaginal glands. The pelvic splanchnic nerve is formed by the parasympathetic nerves from the sacral S2-S4 levels of the spinal cord (preganglionic neurons) (Meston, 2000; Kandeel *et al.*, 2001; McKenna, 2002; Bancroft, 2005; Krüger *et al.*, 2005; Azadzoi & Siroky, 2010; Andersson, 2011; Tezer *et al.*, 2012; Alwaal *et al.*, 2015).

Whereas Reflex Erection arises from direct stimulation of the penis and is mediated by parasympathetic nerves, Psychogenic Erection is mediated by sympathetic nerves. Stimulation of sexual male glands secretion and vaginal secretion is also controlled by parasympathetic nerves. Emission involves the release of sperm through contraction of the gonadal ducts and the sexual glands, and is controlled by sympathetic nerves (the hypograstric nerve is formed by the sympathetic nerves from the thoracic T11 level up to the lumbar L2 level of the spinal cord at preganglionic neurons). Uterus, cervix and vagina contractions are also controlled by sympathetic nerves. Smooth muscle contraction of the vas deferens during ejaculation (release of sperm through the urethra) and vaginal contractions during orgasm, as well as the contractions of the somatic pelvic muscles that accompany the orgasm, are controlled by the sacral spinal nerves (the pudendal nerve is formed by the spinal nerves from the sacral S2-S4 levels of the spinal cord), that in turn convey the genital perineal sensations to the brain (efferent and afferent sensory-motor fibers) (Meston, 2000; Kandeel *et al.*, 2001; McKenna, 2002; Bancroft, 2005; Krüger *et al.*, 2005; Azadzoi & Siroky, 2010; Andersson, 2011; Tezer *et al.*, 2012; Alwaal *et al.*, 2015).

Arousal means a psychological sexual anticipation. Male arousal leads to erection, whereas in females the arousal response is reflected in engorged sexual tissues such as nipples, vulva, clitoris, vaginal walls and vaginal lubrication. Mental stimuli and physical stimuli such as touch, and the internal fluctuation of hormones can influence sexual arousal. Sexual dysfunction includes any problem experienced by either member of the couple during the sexual act that prevents them from having a pleasant and positive sexual experience (Meston, 2000; Kandeel *et al.*, 2001; McKenna, 2002; Bancroft, 2005; Krüger *et al.*, 2005; Azadzoi & Siroky, 2010; Andersson, 2011; Tezer *et al.*, 2012; Alwaal *et al.*, 2015).

In V30M patients, sexual dysfunction is believed to be associated with problems concerning the autonomic nervous system, followed by psychogenic responses. In men, the most common abnormalities related to the reproductive system are erectile dysfunction and retrograde ejaculation. Normally, the urethral sphincter contracts before ejaculation forcing the semen to exit via the urethra. When the sphincter does not function properly, retrograde ejaculation may occur, with semen being introduced into the bladder. The urethral sphincter is composed of smooth muscle and is thus controlled by the autonomic nervous system. The sympathetic nervous system maintains tonic contractions of the urethral muscle, whereas the parasympathetic nervous system relaxes the urethral sphincter muscle during micturition. The parasympathetic activity is responsible for bladder contraction and enables the urethral sphincter to open and urine to be expelled into the urethra. The sympathetic activity leads to contraction and closure of the sphincter. Moderate bladder distension inhibits parasympathetic activity to enable the bladder to be normally filled. When the bladder is full, the afferent activity conveys this information to the brain and this leads to an increase of the parasympathetic tone, together with a decrease in sympathetic activity, causing the urethral sphincter muscle to relax and the bladder to contract, which is followed by micturition (Hita Villaplana *et al.*, 1977; Jung *et al.*, 2012).

In men, erectile dysfunction (controlled by parasympathetic nerves) can be one of the first signs of V30M (Andrade, 1952), and it is also related to the autonomic neuropathy. Problems in ejaculation (controlled by sympathetic nerves) can also be present. Besides retrograde ejaculation (see above) they can also develop secretory azoospermia due to atrophy of the seminiferous tubules, caused by amyloid deposits in the interstitial loose connective tissue around small vessels (Andrade, 1952). These men need psychological support, and there are several drugs that help in erection.

Female Sexual dysfunction affects about 42% of V30M patients. It is due to pelvic nerve injury (neuropathy) caused by destruction of genital myelinated fibers. In this area, amyloid deposition induces nerve loss and decreased blood flow (amyloid causes vessel degeneration) (Alves *et al.*, 1997; Gomes *et al.*, 2011, 2012; Oliveira-e-Silva *et al.*, 2013). Among the symptoms associated with this condition there are reductions on the number of sexual stimuli received by the individual, lubrication deficiencies, lack of sexual desire (due to difficulties in peripheral stimulation) and arousal, painful intercourse, difficulties concerning orgasms and sexual dissatisfaction. More specifically, there is decreased genital area sensation (decreased sexual pleasure stimulation feelings), clitoral blood flow (decreased erection and sensibility; decreased vaginal congestion and dilation), contractile capacity (decreased uterine and vaginal contractions), and glandular secretion (decreased lubrication causes genital discomfort). Recurrent urinary tract infections, vaginal and uterus prolapse, and urinary incontinence - namely coital urinary incontinence (due to detrusor hypo-contractility by loss of parasympathetic nerve stimulation of this smooth muscle of the bladder wall), aggravate the loss of sexual drive (decreased mental arousal), and thus appear associated with dyspareunia (painful intercourse) and sexual dissatisfaction. These patients need psychological support, and there are several pelvic and vaginal therapies that should be introduced, besides surgical corrections of prolapses and incontinence (Gomes *et al.*, 2011, 2012).

Sexual dysfunction is directly associated to disease stage and can be aggravated by the medication taken by the patients. Known medications with a negative impact on sexual function are anti-depressives, anxiolytics, antiepileptics and anti-hypertensives. Affected patients become psychological fragile, as they know they have a potential fatal disease and they are not sexually satisfactory to their partner. This causes loss of sexual motivation as they feel less feminine/masculine, that their body is damaged, unhappy, frustrated, shame, self-image degradation, and failure to meet personal expectations.

Several medications are used to counteract these psychological problems, but on the other hand there are side-effects reflecting on sexual performance. Anxiolytics stimulate gamma-aminobutyric receptors (GABA), and sexual side-effects include decreased libido (decreased gland secretion) and erection (penis and clitoris), which is accomplished through anticholinergic (parasympathetic nerves: erection, gland secretion) and antiadrenergic (sympathetic nerves: orgasm) effects (Tallman *et al.*, 2002). Anti-depression agents increase serotonin activity, which inhibits acetylcholine and norepinephrine receptors, thus inhibiting their functions in arousal, erection and orgasm; they have anticholinergic effects (involved in erection, male and vaginal lubrication); they cause dopamine blockade (dopamine increases sexual arousal and enhance penile erection); nitric oxide inhibition (involved in erection); and increase prolactin secretion (loss of male arousal, erection, orgasm, retrograde ejaculation: hyperprolactinemia impairs luteinizing hormone (LH) release, which causes decreased testosterone production, blocks dopamine actions, increases serotonin secretion and blocks adrenergic receptors) (Baldwin & Mayers, 2003; Buvat, 2003). Antiepileptics are used to treat sensitive disorders and pain, with side-effects causing loss of sexual drive, arousal, erection, gland secretion and orgasm. Gabapentin and Pregabalin are anticonvulsants used to treat neuropathic pain. Although they stimulate GABA actions, their main action is to bind to voltage-gated Ca^2+^ channels of synapses, inhibiting neurotransmitter release in spinal cord neurons (Kaufman & Struck, 2011). Antihypertensives can also cause sexual dysfunction as a side-effect. The effects on erection are supposed to derive from vascular supply impairment and vascular smooth muscle contraction; of adrenergic receptors block (sympathetic nerves: emission and contraction); and cholinergic receptors block (parasympathetic nerves: erection and gland secretion). Some also alter serotonin action (Manolis & Doumas, 2012; Nunes *et al.*, 2012).

### 4. Medical interventions to avoid disease transmission

Current V30M treatments are directed towards relieving specific symptoms, such as pain, infections, cardiovascular problems, kidney failure, bladder dysfunction and genital problems. However, efforts have been made to develop new treatment modalities in order to eradicate or halt the progression of the disease.

#### 4.1. Preimplantation genetic diagnosis

Given that this disease is hereditary and autosomal dominant, each patient has at least one parent suffering from the condition. As the great majority of the carriers are heterozygotes for the disease, they have a 50% chance of passing it on to their children if only one parent is affected or a 75% chance when both parents are affected. Besides social teaching (couple decision to avoid conception), the only way to halt transmission of the disease is the medical termination of pregnancy after a positive Prenatal diagnostic test (Yamashita *et al.*, 2009) or embryo selection after preimplantation genetic diagnosis (PGD) (Carvalho *et al.*, 2001; Almeida *et al.*, 2005; Valdrez *et al.*, 2014).

**Figure 3:**
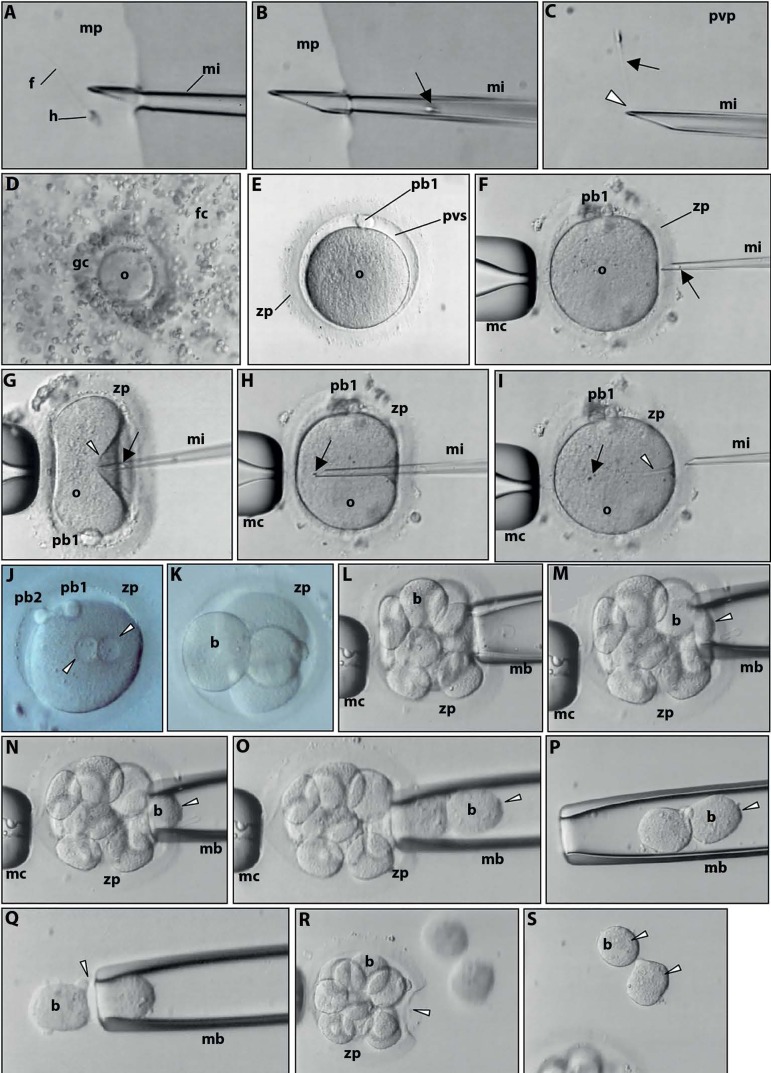
Live images observed at the inverted microscope. Preimplantation genetic diagnosis. A. Selection of a single, morphologically normal spermatozoon (head-h; tail-f) with a 3D forward progressive motility in culture medium (mp) supplemented with PVP to decrease spermatozoon velocity. Microinjection pipette (mi). B. Aspiration of the selected spermatozoon (arrow) into the microinjection pipette (mi). C. Immobilization of the selected spermatozoon (arrow) in PVP (pvp) by crashing (arrowhead) the distal microtubules of the flagellum. D. Cumulus-oocyte complex of the aspirated ovarian follicle. The oocyte (o) is coated by granulosa cells (gc) and encircled by follicular cells (fc) of the cumulus oophorus. E. Mature metaphase II oocyte after denudation of the cumulus and granulosa cells. Note the perivitelline space (pvs), the first polar body (pb1) and the glycoprotein oocyte coat, the zona pellucida (zp) F. For microinjection, the oocyte is held by a contention micropipette (mc) with the first polar body at 3 (F) or 6 (G) o’clock. The microinjection pipette is penetrating the zona pellucida. The spermatozoon is at the distal border of the microinjection pipette, head first (arrow). G. Penetration of the oocyte membrane (arrowhead) by the microinjection pipette. H. The spermatozoon (arrow) is released in the ooplasm. I. Extrusion of the microinjection pipette, leaving the spermatozoon in the ooplasm (arrow). The furrow of the oocyte is still visible (arrowhead). J. The day after microinjection the oocyte shows signs of normal fecundation as revealed by the presence of the second polar body (pb2) and of both pronuclei (arrowheads). K. At the second day after microinjection the zygote cleaved into an embryo with 4 blastomeres (b). L-P. Successive images of the embryo biopsy. Three days after microinjection the embryo has 8 blastomeres. After opening a small hole in the zona pellucida, the embryo biopsy pipette (mb) enters the perivitelline space and gently aspirates two blastomeres (arrowhead). Q. Extrusion (arrowhead) of the two blastomeres to the surrounding medium. R. The embryo is left in culture with the evident zona pellucida hole (arrowhead). S. Note the nuclei of the isolated blastomeres (arrowheads). The diameter of the oocyte is about 110 µm.

The PGD technique enables the detection of the genetic defect in embryos conceived by intracytoplasmic sperm injection, and thus enables to choose the nonaffected embryo for uterine transfer. Preimplantation genetic diagnosis (Figure 3) is performed following a sequence of delicate steps. First, the woman is treated with subcutaneous administration of a gonadotrophin-releasing hormone antagonist, to suppress ovary function. Growth of several early antral follicles is then stimulated with recombinant follicle-stimulating hormone (Pinto *et al.*, 2009). It takes around 8-10 days for the follicles to reach 17mm. When they reach this size, the patient is injected with human chorionic gonadotrophin (HCG) to stimulate the final oocyte maturation and simulate the LH peak. Up to 36 hours after this procedure, follicles are aspirated from the ovary by guided ultrasound. Now, the woman is also given endovaginal progesterone pills to enable endometrium differentiation and induce an implantation window.

Aspirated follicles are incubated for 2h and then cumulus cells are removed by hyaluronidase treatment. After 2h of incubation, denuded oocytes are microinjected with a selected sperm (Tesarik *et al.*, 1994; Tesarik & Sousa, 1995). Sperm are obtained by masturbation (ejaculate), urine collection (in retrograde ejaculation), electroejaculation (in anejaculation) (Barros *et al.*, 1998), or testicular aspiration (in azoospermia or when the quality of sperm retrieved by the other methods is insufficient) (Sousa *et al.*, 2002). After collection, sperm is submitted to a differential gradient centrifugation to select the most morphological normal sperm (simulates vaginal and cervix selection). Then, sperm are incubated in capacitating medium (simulates uterine milieu) and after 1h the most morphological normal and rapid progressive sperm are chosen for injection. Microinjection is performed in an inverted microscope with a heated stage, one spermatozoon per oocyte (Figure 3).

**Figure 4:**
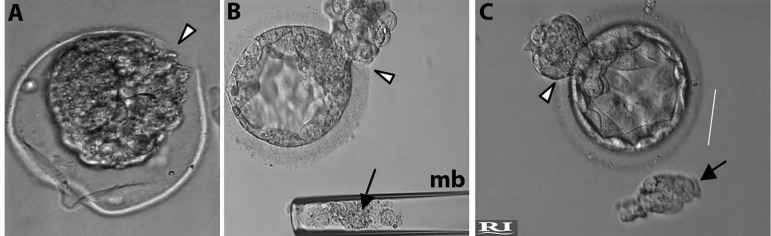
Live images observed at the inverted microscope. Preimplantation genetic diagnosis. Trophectoderm biopsy. A. Note blastocyst retraction and the hole made by the laser beam. B. Note blastocyst expansion and the beginning of hatching (arrowhead). The biopsied cells (arrow) are inside the biopsy micropipette (mb). C. Full blastocyst expansion. Note biopsied cells (arrow) outside the biopsy micropipette.

After microinjection, oocytes are incubated in sequential embryo culture media (simulates tubar and uterine milieu). The day after injection, oocytes are observed to select those which are normally fertilized (presence of two polar bodies and two pronuclei, which indicates oocyte activation and meiosis completion by the oocyte). When embryo development is normal, at day 2 they show 2-4 blastomeres and at day 3 they will present 6-8 cells (Vandervorst *et al.*, 1998). Only day 3 embryos with less than 25% of fragments and with similar sized blastomeres are selected for embryo biopsy. Embryo biopsy is performed in the inverted microscope. A hole on the oocyte zona pellucida is obtained with the assistance of a computer-controlled laser beam. After that, a biopsy micropipette enters the perivitelline space and aspirates 1-2 blastomeres (Figure 3). While the embryo returns to the incubator, each blastomere is transferred to a PCR tube and transported to a Genetic reference laboratory. There, the DNA of each blastomere is isolated and the mutation is screened. After two days, the genetic result is given (Carvalho *et al.*, 2001). At that time (day 5 after fertilization), cultured viable embryos have developed to the blastocyst stage (Gardner *et al.*, 2000). Only those blastocysts with absent mutation are transferred to the uterine cavity. Alternatively, embryo biopsy can be performed at the blastocyst stage (trofectoderme biopsy) and embryos are frozen. After the genetic result, blastocysts are thawed and transferred in a natural cycle. Presently, blastocyst biopsy is the preferable approach in cases when at least four high quality embryos are obtained at day 3 of culture (Figure 4). Pregnancy is evaluated by the rise of serum β-HCG about 12 days after embryo transfer. Overall, the live-birth delivery rate is about 48%.

This procedure has proven to be quite useful for couples affected by the disease and that wish to conceive a healthy child without wanting the termination of pregnancy. These two benefits as well as avoiding the future suffering of the child are the three main reasons that lead V30M carriers to use PGD when conceiving their offspring.

#### 4.2. Medical interventions to improve quality of life

Except for PGD, all other techniques do not avoid the existence of the disease but can merely slow down the production and deposition of the amyloid fibrils in the PNS of patients to improve their quality of life. These include organ transplantation (Furtado, 2000; Sousa *et al.*, 2004; Benson & Kincaid, 2007; Hou *et al.*, 2007; Lobato & Rocha, 2012; Beirão *et al.*, 2012, 2013; Ando *et al.*, 2013; Castaño *et al.*, 2015), stabilizer agents (Tafamidis: prevent TTR from dissociating into monomers) (Coelho *et al.*, 2012, 2013), gene therapy using antisense oligonucleotides (ASO: cause nuclear degradation of the mutated mRNA (Castaño *et al.*, 2015; Niemietz *et al.*, 2015), gene therapy using small interference RNA (siRNA: block translation of the mutated mRNA) (Coelho *et al.*, 2013; Castaño *et al.*, 2015; Niemietz *et al.*, 2015; Wittrup & Lieberman, 2015), and amyloid removers (Castaño *et al.*, 2015). Transplantation did not improve erectile dysfunction but ameliorated urinary incontinence. Clinical trials with Tafamidis, ASO and siRNA revealed a significant delay in the progression of PNS symptoms but data on sexual dysfunction has not been reported. Research is in course to develop synthetic components that will be able to repair the mutated DNA. Only this technique, if administered early in life after screening, would greatly ameliorate quality of life. But to halt the disease as with PGD, it will be necessary to change the germ cell pool, and for now this is only possible in men, as they possess stem cells in the germinal epithelium.

## CONCLUSION

Treatment of V30M remains directed towards relieving specific symptoms in association with liver transplant, molecular and genetic therapeutics. This disease has a substantial clinical impact on reproductive performance, and current treatments do not elicit sexual improvement. Thus, PGD remains the only available technique to eradicate the disease, as it avoids the birth of new patients.
